# Does Attrition during Follow-Up of a Population Cohort Study Inevitably Lead to Biased Estimates of Health Status?

**DOI:** 10.1371/journal.pone.0083948

**Published:** 2013-12-30

**Authors:** Rosie J. Lacey, Kelvin P. Jordan, Peter R. Croft

**Affiliations:** Research Institute for Primary Care & Health Sciences, Keele University, Keele, Staffordshire, United Kingdom; Universität Bochum, Germany

## Abstract

Attrition is a potential source of bias in cohort studies. Although attrition may be inevitable in cohort studies of older people, there is little empirical evidence as to whether bias due to such attrition is also inevitable. Anonymised primary care data, routinely collected in clinical practice and independent of any cohort research study, represents an ideal unselected comparison dataset with which to compare primary care data from consenting responders to a cohort study. Our objective was to use this method as a novel means to assess if (i) responders at follow-up stages in a cohort study remain representative of responders at baseline and (ii) attrition biases estimates of longitudinal associations. We compared primary care consultation morbidities and prescription prevalences among circa 32,000 patients aged 50+ who contribute to an anonymised general practice database (Consultations in Primary Care Archive (CiPCA)) with those from patients aged 50+ in the North Staffordshire Osteoarthritis Project (NorStOP) cohort, United Kingdom (2002–2008; n = 16,159). 8,197 (51%) persons responded to the NorStOP baseline survey and consented to medical record review. 5,121 and 3,311 responded at 3- and 6-year follow-ups. Differences in consulting prevalence of non-musculoskeletal morbidities between NorStOP responders and CiPCA comparison population did not increase over the two follow-up points except for ischaemic heart disease. Differences observed at baseline for osteoarthritis-related consultations were generally unchanged at the two follow-ups (standardised prevalence ratios for osteoarthritis (1.09–1.13) and joint pain (1.12–1.23)). Age and gender adjusted associations between baseline consultation for chronic morbidity and future new osteoarthritis and related consultations were similar in CiPCA (adjusted Hazard Ratio: 1.40; 95% Confidence Interval: 1.34,1.47) and NorStOP 6-year responders (1.32; 1.15,1.51). There was little evidence that responders at follow-ups represented any further selection bias to that present at baseline. Attrition in cohort studies of older people does not inevitably indicate bias.

## Introduction

Cohort studies of health investigate the link between factors measured at baseline and subsequent events in the future. Selective recruitment into a cohort study may result in a difference in prevalence of baseline characteristics between the ‘selected’ cohort and the wider population from which it was derived [1]–[5]. However, simulation studies suggest the validity of associations between baseline exposures and future outcomes is relatively unaffected by such baseline selectivity [6].

Of more potential importance is future loss or drop-out of initially recruited cohort participants (attrition). Although such attrition may be inevitable in cohort studies of older people as health and social difficulties develop with age, there is little empirical evidence as to whether bias due to such attrition is also inevitable - specifically whether (i) responders at follow-up stages in a cohort study remain representative of responders at baseline and (ii) attrition biases estimates of longitudinal associations. If cohort attrition results in data that is missing not at random (MNAR), i.e. the probability of drop-out depends on the outcome of interest and cannot be explained by the observed exposures, then simulation studies indicate this may lead to biased estimates of longitudinal associations [7], [8]. Although complete follow-up of all baseline participants with no attrition is the best protection against possible bias, this is rarely achieved in practice.

Predictors of attrition in cohort studies have often been investigated by comparing the baseline characteristics of those who participate at follow-up with those who drop out [4], [9]–[16]. Other methods have been used to estimate the impact of non-response: some studies have assumed that later responders (e.g. those who respond to the second or third mailings of a questionnaire) are more like non-responders than those who respond to the first mailing [17], whilst others have collected minimum data from those not responding to standard survey reminders [18], [19].

An alternative approach is to directly compare independent data from cohort responders with a comparison population which represents the underlying population from which the cohort was drawn. Routinely collected sources of information on health, such as medical records from primary care or national registers, have been used as comparison populations to assess response bias in cross-sectional studies [5], [20]–[23] and initial selection into a birth cohort study [3]. We report here a novel extension of this methodology, involving routine primary care medical record data from survey responders, which is distinct and separate from their survey data, and an available comparison population, also with routinely collected primary care data, from an anonymised general population medical record dataset which was broader than but included the full populations from which survey participants had been drawn. The comparison is performed at baseline and at each subsequent follow-up stage of the cohort study. In this way, consultation and prescription patterns in cohort responders can be compared with those in the underlying population as an estimate of the presence and extent of attrition bias.

We selected an existing cohort study of older people to test this method empirically. The hypotheses tested were that, if there is no systematic selectivity with respect to health among responders to follow-up in the cohort study, then consultation-recorded morbidity in such responders, collected from routine clinical practice and independently of the cohort research, will not differ from routine consultation morbidity frequency in a larger but comparable general population unselected by study participation, and that associations between record-based baseline measures and record-based outcomes in the two populations will be similar.

## Methods

### Ethics Statement

For the North Staffordshire Osteoarthritis Project (NorStOP), ethical approval was obtained from the North Staffordshire Research Ethics Committee UK and written consent for medical records to be reviewed was given by NorStOP participants. For the Consultations in Primary Care Archive (CiPCA) database, ethical approval was given by the North Staffordshire Research Ethics Committee, UK to download and store anonymised medical record information for research use from participating general practices. All general practices participating in CiPCA inform their patient populations that their anonymised records will be used in this way and all patients are offered the opportunity to withdraw their records from inclusion in CiPCA.

### The Comparison Population

CiPCA is a database of routinely collected and anonymized primary care data from 13 general practices in North Staffordshire, United Kingdom (UK), for which there is established published quality with respect to the completeness and consistency of morbidity recording during all consultations from their registered populations [24]. In the UK, about 98% of persons are registered with a general practice [25] for all routine primary care, and their age-sex registers are considered to be representative of the general population [26].

Annual consultation figures for musculoskeletal conditions drawn from the CiPCA database have been shown to be similar to national databases [27]. Data from the 11 CiPCA practices which contributed data continuously from 2001 to 2008 were included in this study. These 11 practices include 5 practices which participated in NorStOP and which were used for the current analysis.

### The NorStOP Cohort

NorStOP is a prospective cohort study of joint pain and general health in older adults [28]. As part of this cohort study in North Staffordshire, during 2002 and 2003 all people (*n* = 16,159) aged 50 years and over who were registered with five general practices were sent a postal questionnaire which incorporated a range of validated self-report measures regarding joint pain, general health, disability, psychological status and socio-demographics [28]. The questionnaire also asked for consent to view medical records. Electronic consultation and prescribing records for participants consenting to review of their medical records was linked to questionnaire self-report data. In order to limit the possibility of people with joint pain being more likely to take part in the study, the questionnaire was entitled “Health Questionnaire” and the covering letter stated “We are very interested in your reply even if you have not had any pain or other symptoms in the recent past”, although some reference to the study topic was made: “Researchers…are trying to find out about joint pain and other symptoms experienced by people…”. A 3-year follow-up questionnaire was sent in 2005/2006 to those who responded to the baseline survey and were still alive and registered with the practices. A 6-year follow-up questionnaire was sent in 2008/2009. Details of response within the NorStOP cohort at each time point have been published previously [29]–[33].

### The Analysis Design

The objective of the current analysis was to compare consultation morbidity and prescription prevalence obtained anonymously from the routine health care records of the CiPCA comparison population, with the consultation morbidity and prescription prevalence obtained from the routine health care records of NorStOP responders at baseline, at three years and at six years who had consented to use of their medical records. The time periods for each comparison were determined by the timing of the NorStOP surveys: i) the two years prior to the baseline survey; ii) the two years prior to the 3-year follow-up survey; and iii) the two years prior to the 6-year follow-up survey.

Consultations for nine specific morbidities were identified for each time period based on recorded Read codes (see Appendix S1). Read codes are a system of morbidity recording commonly used in UK primary care [34]. Around 95% of consultations with a general practitioner in CiPCA practices are Read coded. Consultations for osteoarthritis (OA) and joint pain were included as this was the main focus of the NorStOP study. Five other chronic problems (ischaemic heart disease, diabetes, chronic obstructive pulmonary disease (COPD), asthma and depression) were included to give an indication of the general health of the participants in NorStOP compared with the CiPCA comparison population. Two acute conditions (otitis media and upper respiratory tract infection (URTI)) were included as markers of general consultation propensity and frequency.

Prescriptions for pain medication were also identified from medical records in each time period. A hierarchical classification of analgesia developed previously [35], [36] was used and prescriptions identified for any pain medication, basic analgesia (e.g. paracetamol, topical non-steroidal anti-inflammatory drugs (NSAIDs)), weak or moderate strength analgesia (e.g. coproxamol, codeine less than 30 mg), strong or very strong analgesia (e.g. codeine 30 mg, morphine) and oral NSAIDs.

For analysis of the comparison population, only patients registered at the practices in CiPCA at both the start and end of that period were included in the analysis. For the first time period (the two years prior to the date of the baseline NorStOP survey), the comparison population in CiPCA consisted of all fully registered patients who were aged 50 years and over at the end of the period. For the second (the two years prior to the 3-year follow-up NorStOP survey) and third (the two years prior to the 6-year follow-up NorStOP survey) time periods, the comparison population were all registered patients aged 53 and over, and aged 56 and over, respectively.

For analysis of the NorStOP responders, analysis of medical records for the first time period was performed on all responders at baseline who consented to record review. For the second period, the analysis was undertaken on the subgroup who also responded at three years. For the third period, analysis was further restricted to those who also responded at six years (Table 1).

**Table 1:** The three time periods and denominator populations for NorStOP and CiPCA, North Staffordshire, UK (2000–2008).

	Period covered	Denominator population	*n*	Age^a^ mean (SD)	Female %
**Time period 1**					
NorStOP	2 years prior tobaseline survey	All baseline surveyresponders consentingto record review	8,197	66.2 (10.06)	53
Comparison (CiPCA)	Calendar years2001–2002	All patients registeredbetween 1/1/2001 and31/12/2002 and aged50 or over at31/12/2002	32,647	65.6 (10.77)	54
**Time period 2**					
NorStOP	2 years prior to1^st^ follow-up survey	All 1^st^ follow-upsurvey respondersconsenting to record review	5,121	67.7 (9.13)	54
Comparison (CiPCA)	Calendar years2004–2005	All patients registeredbetween 1/1/2004 and31/12/2005 and aged53 or over at 31/12/2005	32,830	67.3 (10.02)	54
**Time period 3**					
NorStOP	2 years prior to 2^nd^follow-up survey	All 2^nd^ follow-upsurvey respondersconsenting to record review	3,311	69.3 (8.33)	55
Comparison (CiPCA)	Calendar years2007–2008	All patients registeredbetween 1/1/2007 and31/12/2008 and aged56 or over at 31/12/2008	30,280	69.1 (9.30)	54

NorStOP = North Staffordshire Osteoarthritis Project; CiPCA = Consultations in Primary Care Archive; SD = Standard deviation.

^a^ At end of time period.

### Data Availability

The Research Institute for Primary Care & Health Sciences has established data sharing arrangements to support joint publications and other research collaborations. Applications for access to anonymised data from our research databases are reviewed by the Institute's Data Custodian and Academic Proposals Committee and a decision regarding access to the data is made subject to the National Research Ethics Service Research Ethics Committee’s ethical approval first provided for the study and to new analysis being proposed. Further information on our data sharing procedures can be found on the Institute’s website (http://www.keele.ac.uk/pchs/publications/datasharingresources/) or by emailing the Institute’s data manager (primarycare.datasharing@keele.ac.uk).

### Statistical Analysis

For each time period, the two-year consultation prevalence for both the NorStOP responders and CiPCA comparison population was defined as the number of people with a record of consulting primary care at least once for a morbidity during the relevant two year time period, and is reported per 1,000 persons. 95% confidence intervals (95% CI) were calculated for the consultation prevalences within the NorStOP responder population assuming a Poisson distribution. If the 95% CI included the prevalence for the equivalent time period in the CiPCA comparison population, then this suggested the estimates were similar.

NorStOP responders at each time period were then compared again to the equivalent CiPCA comparison population with respect to consultation prevalence using age and gender standardised prevalence ratios (SPR), the CiPCA comparison population being the standard. A standardised prevalence ratio is the ratio of the prevalences of consultation for a particular morbidity in each of the two populations, standardised for age and gender using indirect standardisation. This analysis was repeated for prescription prevalence based on the number of people prescribed each type of pain medication during a time period, again reported per 1,000 persons.

Finally, to assess whether there was any bias in longitudinal associations identified in NorStOP responders compared to the CiPCA comparison population, the association of chronic morbidity at baseline with a future new record of a consultation for OA or joint pain during the 6-year follow-up was assessed in NorStOP 6-year responders. Chronic morbidity was defined as a consultation for ischaemic heart disease, diabetes, COPD, asthma or depression in the two years prior to baseline survey. Analysis was restricted to those without a consultation for OA or joint pain in the two years prior to the baseline survey. Time from baseline to a new diagnosis of OA or joint pain was identified in the medical records and the association of baseline morbidity with a future diagnosis of OA or joint pain evaluated using Cox proportional hazards regression, adjusted for age and gender. The proportionality assumption was assessed graphically and using Schoenfeld residuals [37], and deemed reasonable for this data. The analysis was then repeated with OA as the sole outcome in those without a record of OA prior to the baseline survey, with further adjustment for a record of joint pain consultation in the two years prior to the baseline survey. These analyses were then also performed in the CiPCA comparison population in those registered for all of 2001 and 2002 (defined for this analysis as the “prior to baseline” period) with follow-up from 2003–2008. Patients in CiPCA were censored at the point of death or leaving the practices.

Analysis was performed using Stata 12.1 for Windows.

## Results

**Figure 1:**
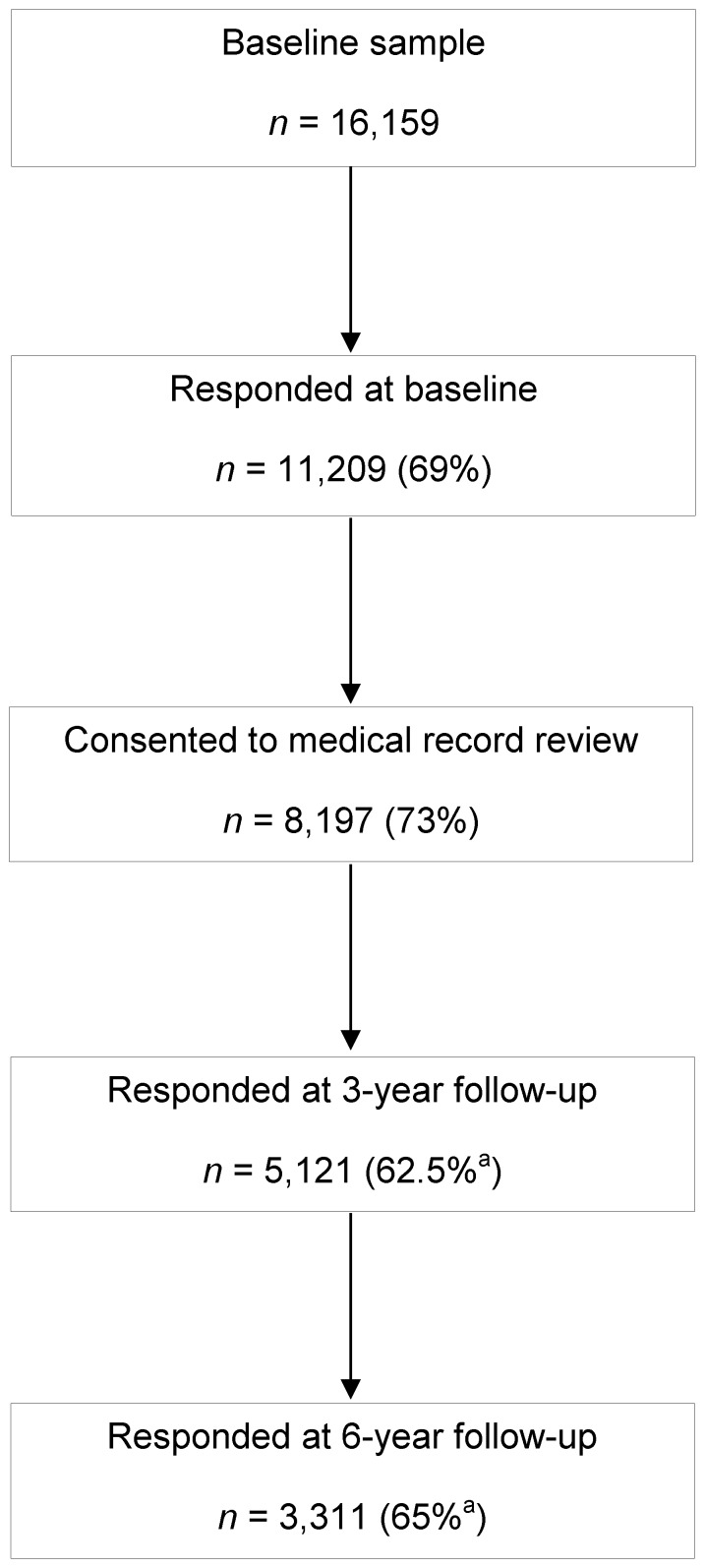
Flow diagram of responders to the North Staffordshire Osteoarthritis Project, United Kingdom (2002–2008). ^a^Unadjusted percentage responding before removing those who had moved or died.

Figure 1 shows the responders at each stage of the NorStOP study. 11,209 responded at baseline. 8,197 of these consented to review of their medical records and form the NorStOP cohort for comparison in this analysis (i.e. 50.7% of the original target survey population). Compared to those who responded but did not consent to record review, consenters were slightly younger (mean 66.2 years vs. 67.4), had a lower proportion who were female (53% vs. 62%) and reported more joint pain (79% vs. 70%). 5,121 (62.5%) of the 8,197 responded at three years. Of the three year responders, 3,311 (65%) responded again at six years. Those who consented to record review constituted 91% of all responders at three years and 92% of responders at six years.

The CiPCA comparison population numbered 32,647 for the first time period, 32,830 for the second time period, and 30,280 for the third time period. Mean age and gender distribution were similar between the comparison population and the NorStOP responders for each time period (Table 1).

Compared with the CiPCA comparison population, NorStOP responders had similar or slightly higher levels of consultation in each time period for diabetes, COPD, asthma, otitis media and URTI with no change in the differences with the comparison population over the two follow-up points (Tables 2 and 3). The comparison population had a slightly higher depression consultation prevalence in the first time period than the NorStOP population. Ischaemic heart disease consultation prevalence was higher in the NorStOP responders than the CiPCA comparison population at 3-year and 6-year follow-ups (SPRs 1.18–1.25).

**Table 2:** Two year consultation and prescription prevalence per 1,000 persons at each survey point in CiPCA comparison population and NorStOP responders.

	Baseline^a^		3 years^b^		6 years^c^	
	Comparison population	NorStOP responders	Comparison population	NorStOP responders	Comparison population	NorStOP responders
	Prevalence	Prevalence(95% CI)	Prevalence	Prevalence(95% CI)	Prevalence	Prevalence(95% CI)
**Consultation prevalence**						
Osteoarthritis	80	93 (86, 100)	77	88 (80, 97)	79	88 (78, 98)
Joint pain	195	220 (210, 231)	206	254 (241, 268)	214	255 (238, 273)
Ischaemic heart disease	84	91 (84, 98)	101	123 (114, 133)	99	127 (115, 140)
Diabetes	63	65 (60, 71)	85	97 (89, 106)	108	111 (100, 123)
COPD	51	57 (52, 62)	49	60 (53, 67)	56	65 (57, 75)
Asthma	50	55 (50, 60)	60	69 (62, 77)	62	70 (61, 79)
Depression	67	51 (47, 57)	58	51 (45, 57)	50	53 (45, 61)
Otitis media	19	21 (18, 25)	15	20 (16, 24)	14	14 (10, 19)
URTI	89	84 (78, 91)	83	94 (85, 102)	88	92 (82, 103)
**Prescription prevalence**						
Any pain medication	521	563 (547, 580)	502	568 (547, 589)	504	545 (521, 571)
Basic analgesia	266	256 (245, 267)	269	265 (251, 279)	297	289 (271, 308)
Weak/moderate analgesia	287	316 (304, 329)	264	313 (298, 329)	253	274 (257, 293)
Strong/very strong analgesia	105	136 (128, 144)	131	159 (148, 170)	168	190 (176, 206)
NSAIDs	207	236 (226, 247)	200	236 (223, 249)	149	185 (171, 200)

NorStOP = North Staffordshire Osteoarthritis Project; CiPCA = Consultations in Primary Care Archive; COPD = Chronic obstructive pulmonary disease; URTI = Upper respiratory tract infection; NSAID = Non-steroidal anti-inflammatory drug; CI = Confidence interval.

^a^ Consultation and prescriptions for the 2 years prior to baseline survey for NorStOP baseline responders; for CiPCA comparison population time period 2001–2002.

^b^ Consultation and prescriptions for the 2 years before 3-year follow-up survey for NorStOP 3-year responders; for CiPCA comparison population time period 2004–2005.

^c^ Consultation and prescriptions for the 2 years before 6-year follow-up survey for NorStOP 6-year responders; for CiPCA comparison population time period 2007–2008.

**Table 3:** Age and gender standardised prevalence ratios (95% CI) comparing NorStOP responders at each survey point to CiPCA comparison population.

	Baseline^a^	3 years^b^	6 years^c^
**Consultation**			
Osteoarthritis	1.13 (1.05, 1.22)	1.12 (1.02, 1.23)	1.09 (0.97, 1.22)
Joint pain	1.12 (1.07, 1.18)	1.23 (1.16, 1.30)	1.18 (1.10, 1.27)
Ischaemic heartdisease	1.03 (0.96, 1.10)	1.18 (1.09, 1.28)	1.25 (1.13, 1.37)
Diabetes	1.00 (0.91, 1.08)	1.10 (1.01, 1.20)	1.01 (0.91, 1.11)
COPD	1.07 (0.97, 1.17)	1.17 (1.04, 1.31)	1.13 (0.98, 1.29)
Asthma	1.10 (1.00, 1.20)	1.15 (1.03, 1.27)	1.10 (0.96, 1.25)
Depression	0.78 (0.71, 0.86)	0.90 (0.79, 1.02)	1.07 (0.91, 1.23)
Otitis media	1.17 (1.00, 1.35)	1.27 (1.04, 1.55)	0.96 (0.70, 1.28)
URTI	0.95 (0.88, 1.02)	1.12 (1.02, 1.23)	1.03 (0.91, 1.15)
**Prescription**			
Any pain medication	1.07 (1.04, 1.10)	1.12 (1.08, 1.16)	1.08 (1.03, 1.13)
Basic analgesia	0.94 (0.90, 0.98)	0.97 (0.92, 1.02)	0.96 (0.90, 1.03)
Weak/moderate analgesia	1.08 (1.04, 1.12)	1.17 (1.11, 1.22)	1.07 (1.00, 1.14)
Strong/very strong analgesia	1.29 (1.21, 1.36)	1.21 (1.13, 1.29)	1.12 (1.04, 1.21)
NSAIDs	1.13 (1.08, 1.18)	1.18 (1.11, 1.25)	1.25 (1.15, 1.35)

NorStOP = North Staffordshire Osteoarthritis Project; CiPCA = Consultations in Primary Care Archive; COPD = Chronic obstructive pulmonary disease; URTI = Upper respiratory tract infection; NSAID = Non-steroidal anti-inflammatory drug; CI = Confidence interval.

For age and gender standardised prevalence ratios, the comparison population (CiPCA) = 1.00.

^a^ Consultation and prescriptions for the 2 years prior to baseline survey.

^b^ Consultation and prescriptions for the 2 years before 3-year follow-up survey.

^c^ Consultation and prescriptions for the 2 years before 6-year follow-up survey.

The two year consultation prevalence of OA was 13% higher for the NorStOP responders than the CiPCA comparison population at baseline, and this difference remained at both follow-up points (SPRs 1.09–1.13 over the three time periods; Table 3). There was a similar pattern, although with a slightly larger difference between NorStOP and the CiPCA comparison population, for joint pain consultation (SPRs 1.12–1.23).

There were consistently slightly higher two year prescription prevalences of any pain medication (SPRs 1.07–1.12) and weak/moderate analgesia (SPRS 1.07–1.17) in NorStOP responders compared with the CiPCA comparison population. NSAID prescription prevalence was 13% higher for NorStOP responders at baseline, increasing to 25% at 6 years but strong analgesia prescription prevalence fell from 29% higher in NorStOP responders at baseline to 12% higher at 6 years (Table 3). Basic analgesics were prescribed to a similar proportion of NorStOP responders and patients in the comparison population (SPRs 0.94–0.97) (Table 3).

The age and gender adjusted risk of future new consultation of OA or joint pain in those with baseline chronic morbidity was similar in the CiPCA comparison population (adjusted HR 1.40; 95% CI 1.34, 1.47) and the NorStOP 6-year responders (1.32; 1.15, 1.51; Table 4). This was also true when outcome was restricted to new diagnosis of OA (comparison adjusted HR 1.25; 95% CI 1.16, 1.34; 6-year responders 1.23; 1.00, 1.52).

**Table 4:** Association of chronic morbidities with new record during follow-up of osteoarthritis, and osteoarthritis or joint pain, in NorStOP and CiPCA.

	Osteoarthritis		Osteoarthritis or joint pain	
	CiPCA comparison population	NorStOP 6-year responders	CiPCA comparison population	NorStOP 6-year responders
Baseline population	32647	3311	32647	3311
No prior record of outcome^a^	30034	3033	24678	2413
Record of outcome during follow-up *n* (%)	3429 (11)	448 (15)	9358 (38)	1151 (48)
Unadjusted HR (95% CI)	1.38 (1.28, 1.48)	1.32 (1.07, 1.62)	1.41 (1.35, 1.47)	1.32 (1.15, 1.51)
Adjusted HR^b^ (95% CI)	1.32 (1.23, 1.42)	1.30 (1.06, 1.60)	1.40 (1.34, 1.47)	1.32 (1.15, 1.51)
Adjusted HR^c^ (95% CI)	1.25 (1.16, 1.34)	1.23 (1.00, 1.52)	–	–

NorStOP = North Staffordshire Osteoarthritis Project; CiPCA = Consultations in Primary Care Archive; COPD = chronic obstructive pulmonary disease; HR = Hazard ratio; CI = Confidence interval.

Chronic morbidity defined as consultation for ischaemic heart disease, diabetes, COPD, asthma or depression prior to baseline.

^a^ No record of i) osteoarthritis and ii) osteoarthritis or joint pain in 2001 or 2002 (comparison) or in the 2 years prior to baseline survey (NorStOP).

^b^ Adjusted for age and gender.

^c^ Adjusted for age, gender and record of joint pain in 2001 or 2002 (comparison) or in the 2 years prior to baseline survey (NorStOP).

## Discussion

This study provides evidence that directly comparing consultation data from cohort responders with that from an available comparison population is a useful method for empirically investigating attrition during follow-up in a cohort study of older people. Our analysis of an existing cohort study shows that attrition did not result in any substantial selection bias at follow-up with respect to routinely recorded morbidities over a six year period. We did, however, find evidence of initial baseline selectivity at cohort recruitment among responders to the baseline survey - baseline participants had consulted more frequently about the topic of the study (OA and joint pain), and had received more and stronger analgesia prescriptions than the comparison population. However, there was little evidence that the cohort responders at follow-up represented any further selectivity with respect to the general population as a whole as represented by the CiPCA comparison population, despite one-third attrition at each stage of the NorStOP cohort follow-up. Furthermore, most other morbidities showed little difference in baseline consultation prevalence between NorStOP cohort responders and the CiPCA comparison population, nor did follow-up cohort responders-to-comparison population morbidity ratios alter in a consistent pattern, apart from the particular example of ischaemic heart disease which became relatively more frequent in the NorStOP population than the comparison population at 6-year follow-up. The latter is the one example in our data where initial selectivity into the cohort (i.e. a higher proportion of people at baseline with OA) may have resulted in additional selectivity at follow-up – a recent prospective study also found an increased risk of ischaemic heart disease among individuals with OA [38]. Finally, analysis of an hypothesised association between baseline chronic morbidities and future outcome (OA or joint pain) was no different in the cohort responders and the comparison population, showing that attrition did not result in biased estimates of the longitudinal associations between the morbidities included and the study outcome.

One explanation for initial selectivity into the cohort was the baseline questionnaire documentation which, although focused on general health, contained cues sufficient to encourage patients “with an interest” in joint pain to participate. Previous studies have found that survey responders with the topic under investigation are more likely to consent to medical record access [39]–[41] or to consult [17]; however, there is also evidence that survey participants do not change their consulting behaviour after completing a health-related questionnaire [42]. By contrast, the evidence for differences in health between participants and non-participants in cohort studies of older adults shows that non-participation at follow-up is more likely in those reporting poorer health [4], [9], [12] and cognitive impairment [4], [10], [11], [43], which provides one explanation of the finding that baseline participants had been less likely to consult about depression. However there is no complete consensus on this issue [11], [44], [45].

The consequence of baseline selectivity means that absolute percentages of people with joint pain and OA and taking analgesia derived from the baseline survey may overestimate rates in the general population. However, this does not affect the main questions we were concerned with in the current analysis: the subsequent impact of attrition during follow-up on the internal validity of longitudinal analysis of the cohort. As long as there is variation in the predictor variable of interest at baseline and sufficient persons are either exposed or not exposed to it to make the outcome of a cohort study viable and powerful enough to detect differences if they exist, then the actual representativeness of the baseline recruited sample of a putative population for a cohort study may not inevitably influence the associations derived from analysis between baseline and subsequent follow-up stages.

Our study has several strengths. Firstly, the existing cohort was a large general population survey of older people with a high response to the questionnaire at each stage. Secondly, although we have previously reported the levels of attrition at each stage of the NorStOP cohort [29]–[33], our novel method of empirically examining the impact of attrition on the selectivity of the followed-up population and on estimates of longitudinal associations now provides evidence that the application of a “follow-up rate criterion” applied to cohort studies may be unreasonable. Thirdly, the quality of CiPCA data is high due to the cycle of training, assessment and feedback undertaken [24] and prevalence of musculoskeletal conditions is comparable to that of larger national general practice databases [27].

A limitation of this study is that we report only on the results of applying our method within one existing cohort. Therefore the absence of major attrition bias found in this study may not be generalisable to other cohort studies with, for example, younger participants, or other morbidities. However, routinely collected primary care data of high quality from the wider general population in countries such as the UK is becoming increasingly available to researchers [46], [47]. Hence, there is the opportunity both to repeat our empirical investigation in other settings and for this method to be used more widely to assess selectivity and bias in cohort studies where consent for linkage to medical records has been obtained and comparison population health care data is available. One practical conclusion from our study is that routinely collected health care data provide one independent source of validation of follow-up in cohort studies and trials. Although such data has not often been available linked to cohort studies in the past, the increasing harnessing and linkage of large health care datasets for epidemiological purposes, for example in cardiovascular disease [48], offer the potential for this to become a more widely available component of cohort study design and validation in the future.

A further limitation is that our analysis is based on cohort responders consenting to medical record review, who were different on some baseline factors such as age and self-reported joint pain to those who responded but did not consent. However, as over 90% of all responders at the two follow-up points consented to record review, they can be considered to fairly reflect the responding population as a whole. In particular, as we have shown before, the possible loss to follow-up from refusal to consent to use of medical records (as distinct from providing replies to questionnaires) does not seem to introduce bias into longitudinal samples [41].

In conclusion, this study provides evidence for a useful method for investigating bias due to attrition in a cohort study of older people and for its potential application to assess selectivity and bias in other cohort studies where consent for linkage to medical records has been obtained and comparison population data is available. Our particular results also contribute empirical evidence that, although the occurrence of attrition in a cohort study should always be investigated as recommended by guidelines such as STROBE (strengthening the reporting of observational studies in epidemiology) [49], attrition in cohort studies of older people may not be an inevitable indicator of selectivity and bias and a flawed result.

## Supporting Information

Appendix S1 **Read codes and terms used for morbidities.** Read codes and terms for the nine specific morbidities included in the study. Read codes are a system of morbidity recording commonly used in UK primary care [34].
(DOCX)
