# Supplementary material for: Does Attrition during Follow-Up of a Population Cohort Study Inevitably Lead to Biased Estimates of Health Status?
Source: PLoS One. 2013 Dec 30;8(12):e83948. doi: 10.1371/journal.pone.0083948 (PMC3875525; doi:10.1371/journal.pone.0083948)
Supplement: Appendix S1 — Read codes and terms used for morbidities. Read codes and terms for the nine specific morbidities included in the study. Read codes are a system of morbidity recording commonly used in UK primary care [34]. (DOCX) [file pone.0083948.s001.docx]

|  | **Read codes starting** | **Read term** |
| --- | --- | --- |
| Ischaemic heart disease | G3… | Ischaemic heart disease |
| Diabetes | C10… | Diabetes mellitus |
| Chronic obstructive pulmonary disease | H3... | Chronic obstructive pulmonary disease |
|  | (excluding H33.. | Asthma |
|  | H35.. | Extrinsic allergic alveolitis) |
| Asthma | H33.. | Asthma |
| Depression | E2B.. | Depressive disorder NEC |
|  | Eu32. | [X]Depressive episode |
|  | E2003 | Anxiety with depression |
| Otitis media | F51.. | Nonsuppurative otitis media + eustachian tube disorders |
|  | F52.. | Suppurative and unspecified otitis media |
| Osteoarthritis | N05.. | Osteoarthritis and allied disorders |
| Joint pain | N094. | Pain in joint – arthralgia |
|  | N245. | Pain in limb |
| Upper respiratory tract infections | H02.. | Acute pharyngitis |
|  | H05.. | Other acute upper respiratory infections |
